# Infantile Paralysis

**Published:** 1891-06

**Authors:** A. Van Hoff Gosweiler

**Affiliations:** Baltimore.


ARTICLE III.

### INFANTILE PARALYSIS.

BY A. VAN HOFF GOSWEILER, A.M., M.D., OF BALTIMORE.

[Read before the Medical and Surgical Society of Baltimore, June 11, 1891.

Through improved methods of investigation and advanced knowledge, the location of the lesion in the spinal cord in infantile paralysis has of late years been ascertained —the view that the disease is an "essential" affection of the peripheral nerves or of the muscles is incorrect. This generic name is unfortunate and misleading, for it is not, as the name would imply, the only form of paralysis that occurs in children, and even if it were, it is not confined to the period of infancy, but attacks persons of any age. It is, however, akin to a form of paralysis that is by no means uncommon in adults, to which M. Duchenne *de Boulogne*, of France, has applied the name, progressive muscular atrophy. Neither does the name, infantile spinal paralysis, describe it, as spinal paralysis in children may arise from spinal meningitis, tumors, pressure-myelitis in Pott's disease of the spine, acquired diphtheria, syphilis, or other causes. M. Duchenne recognized it first in 1849, and brought it before the profession in 1853, calling it infantile atrophic paralysis. "If I were to give this disease an anatomical name, I should call it acute paralysis of childhood from fatty atrophy of the anterior spinal cells." M. Duchenne thus asserts his belief that its origin is spinal, although no post-mortem examinations had been made to confirm it.

It was not until 1830, that J. von Hein first described its clinical features; in 1863, Cornil microscopically observed distinct alterations in the cord; in 1865, Prevost, Vulpian, Laborde, Erb, Leyden, located, and in 1868, J.

Lockhart Clark confirmed, the essential lesion in the anterior horns of gray matter in the cord; but it remained for Charcot and Joffroy in 1870, to point out the degeneration as well as the constancy of the lesions, and not until then can it be said that its pathology began to be understood.

Though infantile paralysis, or poliomyelitis anterior acuta infantilis,—a name proposed by Professor Kussmaul, indeed, preferable in that it describes the pathology of the disease, a true poliomyelitis—is usually an obscure, warm-weather spinal disease, it has been observed coming on suddenly, but seldom after the age of four years. According to Gowers, of all cases under ten years, three-fifths occur in the first two years of life, and, he says, there is little doubt that a considerable number of cases are congenital.

Dr. T. G. Morton, *PhiladelphiaMedical News*, July 12, 1890, believes that most cases of congenital club-foot are the result of an intra-uterine paralysis, for in all palsied muscles were found. One of M. Duchenne's cases was affected twelve days after birth; Bramwell's in three weeks; Wharton Sinkler's 345 cases in the Philadelphia Infirmary for Nervous Diseases, lately published in statistical form, 135 below two years, and 56 under one.

It is claimed that the paralysis in typical cases is ushered in with fever and restlessness. Charcot regards the fever as the usual precursor, and most of the textbooks follow his example; but in the cases I have seen, I have failed to obtain any history of high temperature. West lays little stress on the initial fever, making it rather the exception than the rule.

Apart from the febrile initial stage and the sudden onset of paralysis, it occurs to the discriminative diagnostician that the uniformity of the lesion in cervical and lumbar enlargements of the spinal cord, the invariable immunity of the sensory functions and of the visceral sphincters, the rapid disappearance of the reactions of the muscles to the faradic electric current, the early atrophy, the fall of temperature, finally, the deformity,—that all these phenomena are found combined in no other disease. In fact, it cannot be denied that peripheral paralysis of single limb—of one arm or leg—may resemble in its clinical characters the centric affection which we are considering. However, the absence of anæsthesia, of a characteristic decubitus, of paralysis of the sphincters, distinguish it from acute, central, transverse myelitis, multiple neuritis or diphtheritic paralysis. From the effects of injuries, especially from stretching or compression of a nerve-trunk and congenital dislocation of the shoulder-joint, we learn in the matter of differential diagnosis, that paralysis may arise and be accompanied after a short time, by atrophy of the muscles and loss of their reactions to faradic electricity, just in certain cases, reported by Charcot, of peripheral paralysis of the facial nerve.

Statistics inform us that in more than half the cases the lower limbs are affected; of the remainder, the majority represent implications of the arms, notably the deltoid muscles (palsy of Erb), and legs, or, perhaps, arm and leg, and very seldom the upper extremity alone.

All investigators assign some cause, such as teething, cold or damp, injuries to the spine, measles, scarlatina, malarial or other fever, convulsions or concussion; but when such a variety of wholly different causes are assigned, which posses no feature in common, we are warranted in thinking they are merely concomitants or accidents. Paralysis in infants following a chill, when the body is heated, gives rise to suspicion of poliomyelitis anterior. Heredity has, perhaps, a distinct influence in the production of the disease, but it is only after a popular mode of expression that we can consider it as a cause. At the Fourth Congress of Russian physicians, recently held, Dr. Rot made a communication, declaring that heredity is the only etiological factor that has been proven, "the primary cause of the affection must be sought for in the modifications of that part of the fecundated ovum, which enters into the formation of the nervous system." Gowers and Buzzard seem quite paradoxical, if not right, in their statements, the one being strongly impressed with its heredity; the other believing that "it is more common than not, for the disease to attack fine, grown, hearty children, for neuropathic heredity generally does not materially impress itself in an apparent manner upon the nutrition or growth of infantile life." During the period of dentition, children are liable to disorders of the cerebro-spinal system, and, as from apparently slight causes, we find convulsions the cause of the death of numberless infants, seemingly robust; so we see in this affection, as little cause producing paralysis.

Of course, we have loss of heat aad atrophy in the muscles of the affected limbs; but what ls the cause of the atrophy? Is it due merely to their not being called into action? or is the atrophy a feature of the disease as the paralysis and dependent upon the morbid changes in the nerve-centres? The latter seems most probable, as we observe the atrophy extends to the bony system, the nutrition of which is involved. Evidently, this atrophic degeneration, if not inherent, is a real sequence of inflammatory process in the cord. This suggests the question of the utility of topical remedies, sûch as rubbing, muscle-beating, massage, perhaps electricity.

Here, again, in the muscle-lesion, we observe a marked contrast to the order of sequences that obtain in the cognate disease, progressive muscular atrophy, for while in the former, the paralysis always precedes the atrophy; in the latter, the atrophy precedes the paralysis, and determines the amount. If not an anomaly, perhaps a problem for the physician, why the affected limbs in the latter react normally to electrical stimuli, and in the former, galvanic reaction is either wholly or partially lost?

It is a characteristic point that the paralysis almost always reaches its worst at the very beginning, as in the apoplectic paralysis of adults, or in the first twenty-four or forty-eight hours. After that there is a marked improvement. The power of motion is rapidly recovered. After some weeks the paralysis is often confined to a single group of muscles in one arm or leg with persistence; in other cases the symptoms suddenly improve. Occasionally, after a week, some of the muscles contract, but feebly, others not at all, to the faradic electric current. "This is," says Prof. Henoch, of Berlin, "a bad sign, for when the muscles cease to react some weeks after the onset of the disease, they usually remain incapable to reaction to the electric current during the whole of life. The paralysis and atrophy may be very well marked, and yet the limbs scarcely appear shortened, and the growth of the bone may be arrested to considerable extent."

When called in time, we are first to combat the congestion and inflammation, which, manifestly, are the conditions of the gray horns of the spinal cord; hence we have a clue to the therapeusis we should employ. At this early stage we should ignore vigorous faradic stimulation, either peripheral or from center to periphery, for it will exhaust motor excitability; but should adopt, in particularly adapted cases, the mild, galvanic, uninterrupted current, to be sent down through the injured cord, out through the nerves to the flabby muscles. However, when the damaged motor centres can bear later peripheral excitation, the faradic, the induced static, or franklinic interrupted current may be employed. After the eighth day Simon, of Paris, uses a weak galvanic current, applying the positive pole to the shoulder and arm, the negative pole being placed in a basin of water, in which the child's hand rests. The sitting should never last more than eight minutes. The functions of the skin may then be stimulated with salt and sulphur baths. In the early stage Dr. Althaus advises the injection of ergotine, ¼ gr., for a child a year old, in order to contract the arterioles of the part, to deplete its blood supply. He stimulates the muscles as they become affected with injections of strychnine. Conium and chloral may be used to calm nervous excitement. Dr. E. C. Segum recommends counter-irritation over the spine, bromides, and arsenic, while others use cupping, leeches, and iodide of potassium. Brown-Sequard has well encouraged us to employ belladonna, in that it is capable of controlling the inflammatory process in the cord. Prof. Fraser, of Edinburgh, has confirmed this claim. The same indication that calls for rest to the cord indicates abstinence from large doses of strychnine and electricity. If pain or fever are present, use ether spray to the spine, ice, gelsemium, aconite, antipyrine, internally.

All cases do not get well under treatment, but enough recoveries occur to give hope for a bright future for these little ones, when the family physician will either treat them correctly or send them to the neurologist, who will take the necessary and timely pains with them. General medicine made nervous diseases an opprobrium until neurology grew into an important specialty, and gave hope to thousands, as it has given to these little patients, who are too late and often left to the tender care of modest prophets to grow up in some instances, needlessly deformed.—*The Times and Register.*

